# Mental health and wellbeing among people with informal caring responsibilities across different time points during the COVID-19 pandemic: a population-based propensity score matching analysis

**DOI:** 10.1177/17579139221104973

**Published:** 2022-07-05

**Authors:** Hei Wan Mak, Feifei Bu, Daisy Fancourt

**Affiliations:** Department of Behavioural Science and Health, University College London, London, UK; Department of Behavioural Science and Health, University College London, London, UK; Department of Behavioural Science and Health, University College London, 1–19 Torrington Place, London WC1E 7HB, UK

**Keywords:** informal carers, COVID-19, mental health, mental wellbeing

## Abstract

**Aims:**

Due to a prolonged period of national and regional lockdown measures during the coronavirus (COVID-19) pandemic, there has been an increase reliance on informal care for informal carers. In light of this, the current study compared the experiences of carers and non-carers on various mental health and wellbeing measures across six key time points during the pandemic.

**Methods:**

Data analysed were from the University College London (UCL) COVID -19 Social Study. Our study focused on six time points in England: (1) the first national lockdown (March–April 2020); (2) the beginning of first lockdown rules easing (May 2020); (3) the second national lockdown (November 2020); (4) the third national lockdown (January 2021); (5) the easing of the third lockdown (March 2021); and (6) the end of restrictions (July–August 2021). We considered five mental health and wellbeing measures: depressive symptoms, anxiety symptoms, loneliness, life satisfaction, and sense of being worthwhile. Propensity score matching was applied for the analyses.

**Results:**

We found that informal carers experienced higher levels of depressive and anxiety symptoms than non-carers across much of the pandemic. During the first national lockdown, carers also experienced a higher sense of life being worthwhile. No association was found between informal caring responsibilities and levels of loneliness and life satisfaction.

**Conclusion:**

Given that carers are an essential national healthcare support, especially during a pandemic, it is crucial to integrate carers’ needs into healthcare planning and delivery. These results highlight that there is a pressing need to provide adequate and targeted mental health support for carers during and following this pandemic.

## Introduction

Prior to the COVID-19 pandemic, there were around one in eight adults (approximately 6.5 million people) providing some form of informal care in the UK, estimated to have a replacement value of £132 billion a year.^1^ Informal care is defined as unpaid care and support for others (typically family, relatives, friends, or neighbours) who may have a disability, chronic illness, mental health problem, or other care needs. This can include providing supervision, practical or instrumental care (e.g. shopping, household chores) and personal care (e.g. dressing, bathing, eating, using the bathroom, emotional support).^2,3^ With population ageing where the life expectancy for people with long-term health conditions has improved, the demand for informal care has increased to meet the needs and to support the sustainability of health and social care system.^3^ As such, informal care is becoming increasingly important within society.

However, informal care, especially personal care, can be physically and mentally demanding. According to Carers UK,^1,4^ nearly one in seven of informal carers juggle their caring responsibilities with work, 15% provide over 50 h of care per week, and 17% care for more than one person. In addition, 3% of the UK general population (more than 1.3 million people) are ‘sandwich’ carers – people with the dual responsibility of caring for elderly or disabled/sick family members and young children. Often, carers are faced with challenging tasks and stressful situations and are required to maintain high levels of vigilance; this can create chronic stress.^5^ This can have a profound impact on carers’ personal and social life, and physical and mental health and wellbeing. A substantial wealth of literature shows that caring responsibilities have an adverse effect on physical and mental health and health-related behaviours. For instance, it has been shown that people who provide informal care experience higher levels of depression and anxiety, inadequate sleep, higher levels of loneliness, and a higher risk of stroke.^1–3,6–8^ However, there are also some reported benefits of caregiving, such as self-esteem and sense of meaning.^9–11^

During the coronavirus (COVID-19) pandemic, members of the public faced prolonged periods of social distancing, reduced access to local services and community facilities, and restricted face-to-face contacts. Particularly, people considered clinically vulnerable (e.g. older adults aged 70 or above and people with specific medical conditions) faced greatest social restrictions as they were advised to follow stricter advice, often not leaving their homes (‘shielding’). For many, this led to an increased reliance on informal care and a consequent increase in care intensity for informal carers.^12^ Indeed, a report from Carers UK has shown that there were an additional 4.5 million informal carers during 2020 while the outbreak of COVID-19 was ongoing.^13^ Also, limited access to health services means that many carers faced more stressful situations related to care recipients’ medical conditions.^14,15^ Moreover, to protect those they were caring for, carers themselves had to shield, facing the same tougher restrictions on their social lives and disrupting usual social support networks. There are, consequently, concerns that the mental health of carers was adversely affected during the pandemic. However, while there has been wide-spread concern for the negative impact of COVID-19 pandemic on mental health of the public^16–19^ and formal carers and other healthcare professionals,^20,21^ with results suggesting worsening mental health during the pandemic compared with before, less attention has been paid to the mental health and wellbeing of informal carers during the pandemic.^12–15,22–24^

Among the studies that have been conducted, it has been shown that, since the start of the pandemic, people who provided informal care were likely to be women, younger adults, have children under the age of 18, and have paid work.^13^ These individuals often experienced a double burden of working or childcare and providing informal care. Some preliminary research has already shown the negative impacts of the pandemic on informal carers. These include increased levels of depression (especially for those who spend 20 h or more per week on caring),^14^ increased mental strain (e.g. the concerns of risk of COVID-19 infection in family),^23^ increased alcohol consumption and use of illegal drugs,^22^ increased feelings of frustration,^24^ and feelings of loss of control and uncertainty.^12^ However, to date these studies have generally relied on relatively small sample sizes and focused on one time point rather than looking at the evolution of experiences across the pandemic. Furthermore, there has been little research on the impact of informal caring on positive wellbeing during the pandemic.

In light of this, the present study compared the experiences of carers and non-carers on a number of mental health and wellbeing measures, namely depressive symptoms, anxiety symptoms, loneliness, life satisfaction, and a sense that life is worthwhile across various time points during the COVID-19 pandemic. As caring responsibilities are socially patterned, with the demographics of carers (e.g. females)^1^ already linked to less favourable mental health and wellbeing outcomes, this study aimed specifically to disentangle whether the negative impacts of informal caring responsibilities on carers’ mental health and wellbeing were attributable to individual demographics or the role of being an informal carer itself. While direct experimental studies in this context were not feasible or practical, we sought to mimic experimental conditions and to effectively account for the effects of observed confounding factors by using the statistical technique of propensity score matching (PSM).

## Methods

### Participants

This study analysed data from the UK COVID-19 Social Study run by University College London (UCL), a longitudinal study that focuses on the psychological and social experiences of adults living in the UK during the COVID-19 pandemic. The study commenced on 21 March 2020 and involves regular online data collection from participants for the duration of the pandemic. The study is not random and therefore is not representative of the UK population. However, it does contain a heterogeneous sample that was recruited using three primary approaches. First, convenience sampling was used, including promoting the study through existing networks and mailing lists (including large databases of adults who had previously consented to be involved in health research across the UK), print and digital media coverage, and social media. Second, more targeted recruitment was undertaken focusing on (1) individuals from a low-income background, (2) individuals with no or few educational qualifications, and (3) individuals who were unemployed. Third, the study was promoted via partnerships with third sector organisations to vulnerable groups, including adults with pre-existing mental health conditions, older adults, carers, and people experiencing domestic violence or abuse. The study was approved by the UCL Research Ethics Committee [12467/005] and all participants gave informed consent. A full protocol for the study is available online at https://github.com/UCL-BSH/CSSUserGuide.

This study focused on mental health and wellbeing among respondents with caring responsibilities across sic key time points during the pandemic. Given that there were variations in rules and restrictions and the time points that changes to these rules came in across different nations in the UK, we only considered participants who lived in England. We also restricted our sample to participants who completed the survey within 7 days of each time point to correspond to changes in the study design. (At the early stage of the study, participants were followed-up weekly. In August 2020, the study was converted to monthly follow-up and participants were randomly assigned into 4 groups receiving the survey link at different weeks). We further restricted our sample to those who provided responses to all measures. Participants who opted not to provide details on their demographic background (e.g. gender and household income) were additionally excluded from the analysis. Specifically, our six time points were the 5–7 days following the introduction of each of these measures: (1) the first national lockdown (data captured 28 March – 3 April 2020; *N* = 10,414); (2) the beginning of first lockdown rules easing (data captured 16–22 May 2020; *N* = 19,259); (3) the second national lockdown (data captured 14–20 November 2020; *N* = 3,712); (4) the third national lockdown (data captured 16–22 January 2021; *N* = 3,408); (5) the easing of the third lockdown (data captured 20–26 March 2021; *N* = 4,068); and (6) the end of restrictions (data captured 31 July – 6 August 2021; *N* = 3,128).

### Measures

#### Caring responsibilities

Participants were asked whether they had caring responsibilities for elderly relatives or friends, people with long-term conditions or disabilities, or grandchildren. A binary variable was created to indicate if they had any of the responsibilities.

#### Outcome variables

Five mental health and wellbeing variables were considered. *Depressive symptoms* was measured using the Patient Health Questionnaire (PHQ-9), a standard instrument for diagnosing depression in primary care which consists of nine items with 4-point responses ranging from ‘not at all’ to ‘nearly every day’.^25^ Higher overall scores indicate more depressive symptoms. *Anxiety symptoms* was measured using the Generalised Anxiety Disorder assessment (GAD-7), a well-validated tool used to screen and diagnose generalised anxiety disorder in clinical practice and research.^26^ The assessment includes seven items with 4-point responses ranging from ‘not at all’ to ‘nearly every day’, with higher overall scores indicating more symptoms of anxiety. *Loneliness* was measured using the three-item UCLA-3 loneliness, a short form of the Revised UCLA Loneliness Scale (UCLA-R).^27^ Each item is rated with a 4-point rating scale, ranging from ‘never’ to ‘always’, with higher scores indicating greater loneliness. *Life satisfaction* was measured using the Office for National Statistics (ONS) personal wellbeing question ‘overall, how satisfied are you with your life nowadays?’, a 10-point scale. *Sense of that life is worthwhile was* measured using the ONS personal wellbeing question ‘overall, to what extent do you feel the things you do in your life are worthwhile?’, a 10-point scale.^28^ For both ONS scales, higher scores indicate higher levels of life satisfaction or sense of being worthwhile.

#### Covariates

This study considered a set of covariates that could be associated with both caring responsibilities and/or mental health/wellbeing outcomes based on previous empirical research.^29,30^ These included age groups (age 18–29, 30–59, 60+), gender (male versus female), ethnicity (white versus ethnic minorities), living arrangement (living alone, not living alone and not living with children, not living alone and living with children), marital status (married/in a relationship versus not married/not in a relationship), education (degree or above versus without a degree), employment status (employed versus not employed), household income (<£30,000 versus ⩾£30,000 per annum), keyworker status (yes versus no), living area (city/town versus remote area, e.g. village/hamlet/isolated dwelling), long-term mental/physical health condition (yes versus no), having minor/major stress about COVID-19 (yes versus no), and confirmed/suspected of contracting the COVID-19 virus (yes versus no).

We also considered perceived social support and empathy. For perceived social support, it was measured using an adapted version of the six-item short form of Perceived Social Support Questionnaire (F-SozU K-6). Each item is rated on a 5-point scale from ‘not true at all’ to ‘very true’. Minor adaptations were made to the language in the scale to make it relevant to experiences during COVID-19 (Supplementary Table 1). Higher scores indicate greater perceived social support.^31,32^ For empathy, it was measured using the Interpersonal Reactivity Index (IRI). Two scales were the focus in the COVID-19 Social Study: empathetic concern/‘emotional empathy’ and perspective-taking/‘cognitive empathy’. Both scales consist of seven items with a 5-point measure ranging from ‘does not describe me well’ to ‘describe me very well’, and were averaged. Higher scores indicate greater levels of empathetic concern or perspective-taking.

### Statistics

Our analysis used PSM, a technique that stimulates an experimental setting in an observational dataset and creates a treatment group and a control group from the sample.^33^ One advantage of using PSM over regression approaches is that it controls more effectively for the effects of observed confounders, and hence while results remain observational, bias attributable to confounding can be minimalised significantly. We used PSM to estimate the average treatment effect for the treated (ATT), which is the difference between the average mental health/wellbeing outcomes of participants who had caring responsibilities (carers) and the average outcomes for the same group under the hypothetical scenario that they did not have any caring responsibilities (non-carers).

In the analysis, we used weighted PSM models and applied the kernel matching method with cross-validation bandwidth.^34^ Kernel matching uses weighted averages of all individuals in the control group to create the counterfactual outcome, and matches participants in the treatment group to those in the control groups based on the distance of their propensity score. Higher weight is given to the matches whose propensity scores are closer to each other and lower weight to those whose propensity scores are distal from each other.^35^ A common support condition was imposed to ensure the quality of the matches;^30^ only less than 2% of the data were dropped (mostly from the control units). Regression adjustment was also applied on the matched sample to reduce bias due to residual differences after matching and to obtain an unbiased estimate of the treatment effect.^34,36,37^ Missing values were handled with list-wise deletion. High quality of matching was achieved. As shown in Supplementary Figures 1–6, the density distributions of the treatment and control groups overlapped across two study samples across the six time points, indicating good balances of the observed variables between the groups after matching. This suggests that the confounding bias relating to observed covariates should have been reduced significantly.

In addition to the main analysis, three sets of sensitivity analysis were performed. First, we compared mental health and wellbeing between carers and non-carers by restricting the sample to those who reported that their mental health had got worse during the first lockdown in April/May versus before the pandemic. Analysing this would shed light into whether carers continued to suffer more mentally compared to those who were not carers at a time when the mental health of the whole UK population had declined.^19^ Second, we tested whether caring intensity may play a role in affecting informal carers’ mental health and wellbeing. Two binary variables were generated, with one using 3 h or above as the threshold (3 h or above versus less than 3 h) and a higher intensity threshold (6 h or above versus less than 6 h). Due to data availability, we were only able to test the intensity in the first two time points: the first national lockdown and the easing of the first lockdown.

To account for the non-random nature of the sample, all analyses were weighted to the proportions of gender, age, ethnicity, and education obtained from the Office for National Statistics.^38^ All analyses were carried out using Stata/MP 17.0.

## Results

### Descriptive statistics

In our analytical samples across six time points, around one in four self-identified as informal carers (in line with the Carers Week 2020 report).^13^ While the samples shared very similar backgrounds, there was some heterogeneity especially between the first and final time points. For instance, there were fewer younger adults and slightly more older adults aged 60+ in the final time point. Also, there was a decline in stress about COVID-19 and in confirmed or suspected COVID-19 cases as the pandemic continued. Respondents’ mental health and wellbeing, on the other hand, were fairly stable (Supplementary Table 2).

**Figure 1:**
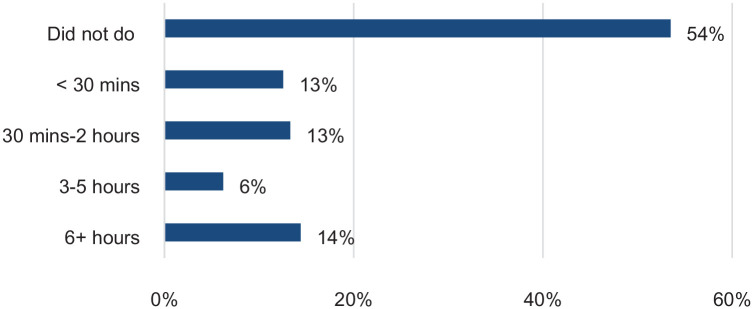
Time spent on caring for a friend or a relative in a day among informal carers Source: UCL Covid-19 Social Study.

Among respondents who provided informal care, when asked to report on the last weekday, 54% reported of not caring for a friend or a relative (suggesting that caring duties were not full-time for half of the sample), more than one in four reported spending 2 h or less on caring, and one in five reported spending 3 or more hours (Figure 1).

### Depressive symptoms

**Figure 2:**
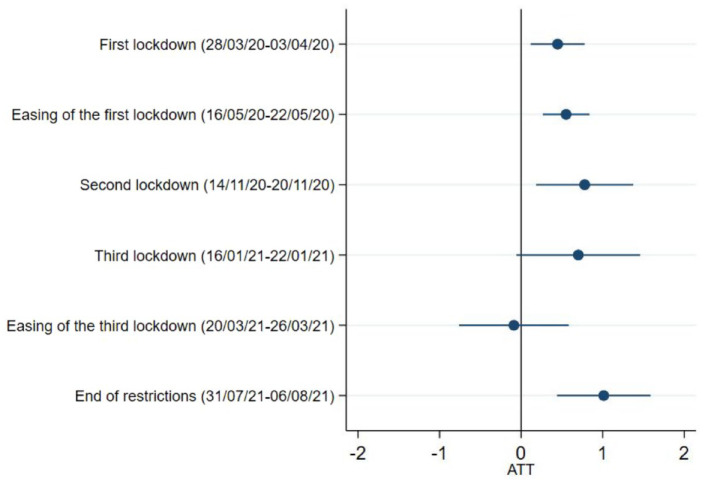
Depressive symptoms

Our results show that carers had more depressive symptoms than non-carers during the first national lockdown, easing of the first lockdown, the second national lockdown, and the end of restrictions. The estimated average treatment effect of being carers on the levels of depression appeared to be the strongest when all the restrictions were lifted in July 2021 (ATT = 1.01, 95% CI = 0.44,1.59) (Figure 2 and Supplementary Table 3). No differences were seen during third lockdown or its easing.

### Anxiety symptoms

**Figure 3:**
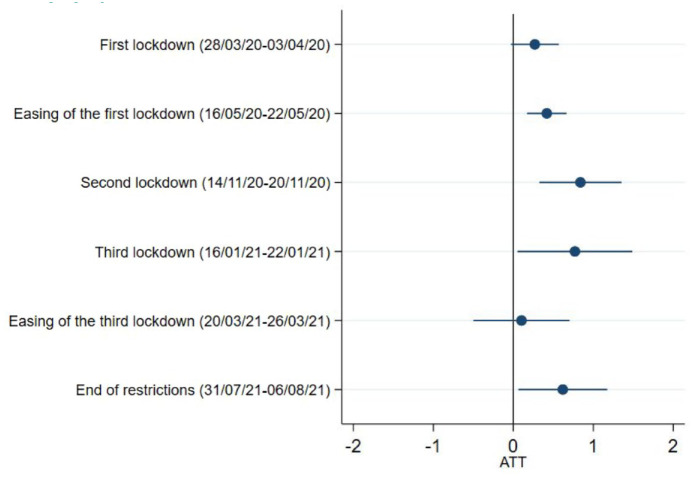
Anxiety symptoms

There were no meaningful differences between carers and non-carers during the first lockdown in anxiety (ATT = 0.27, 95%CI = 0.03,0.57). However, similar to depressive symptoms, we found that caring responsibilities were associated with higher levels of anxiety during the easing of the first lockdown, the second and third national lockdowns, and the end of restrictions. The estimated treatment effect of being carers on the anxiety levels were the strongest during the second lockdown in November 2020 (ATT = 0.84, 95%CI = 0.33,1.35), and were the modest when the first national lockdown began to ease (ATT = 0.42, 95%CI = 0.17,0.67) (Figure 3 and Supplementary Table 3). There were no differences during the easing of third lockdown.

### Loneliness

**Figure 4:**
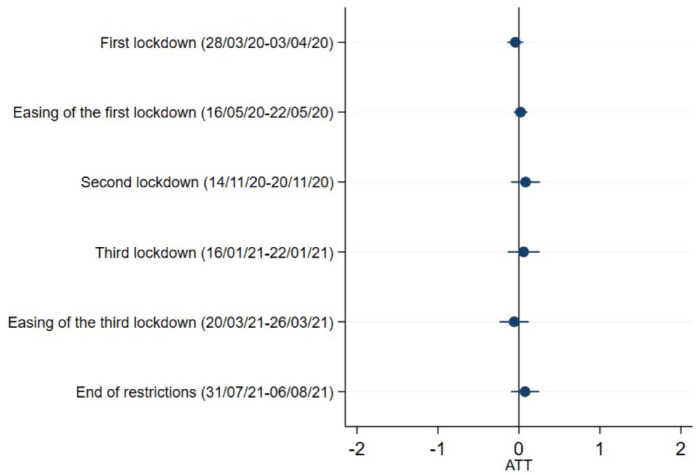
Loneliness

No association was found between caring responsibilities and the levels of loneliness at any of the time points (Figure 4 and Supplementary Table 3).

### Life satisfaction

**Figure 5:**
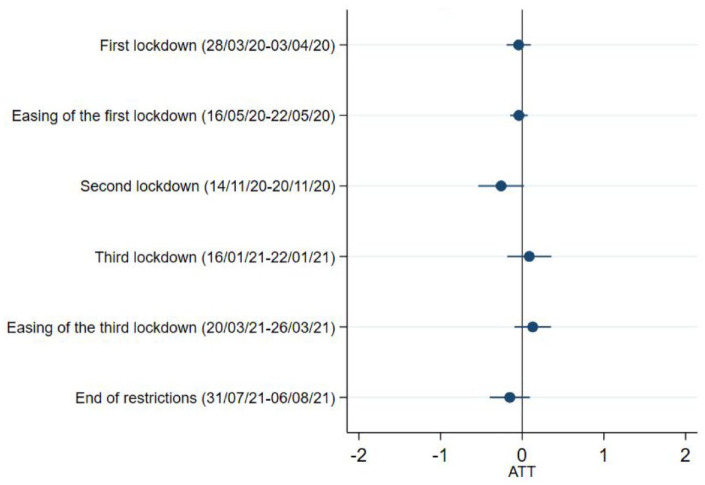
Life satisfaction

No association was found between caring responsibilities and the levels of life satisfaction at any of the time points (Figure 5 and Supplementary Table 3).

### Sense of being worthwhile

**Figure 6:**
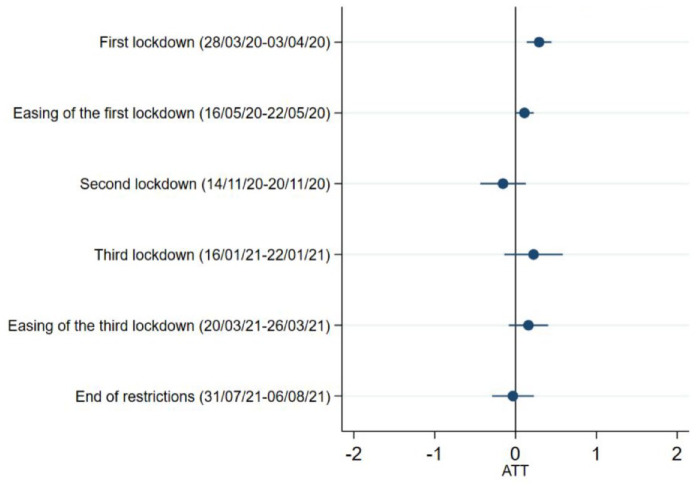
Sense of being worthwhile

Our analysis shows that respondents with caring responsibilities were more likely to have a higher sense of life being worthwhile, but only during the first national lockdown in March 2020 (ATT = 0.29, 95%CI = 0.14,0.44) (Figure 6 and Supplementary Table 3).

### Sensitivity analysis

When restricting our analyses just to respondents who experienced a decline in their mental health during the first lockdown compared to prepandemic periods, informal carers were more likely to experience higher levels of depressive symptoms when all restrictions were lifted in July/August 2021 (ATT = 1.93, 95%CI = 0.51,3.35). No associations were found for other outcomes, nor for other time points (Supplementary Table 4). When comparing various levels of intensity, we found that informal carers who cared for 3 or more hours a day experienced greater levels of anxiety during the easing of the first lockdown compared to those who cared for less than 3 h (ATT = 0.69, 95%CI = 0.10,1.28) (Supplementary Table 5a). Results were consistent when comparing those who cared for 6 or more hours a day versus those who cared for less than 6 h (ATT = 0.72, 95%CI = 0.05,1.38) (Supplementary Table 5b).

## Discussion

This study examined the differences in mental health and wellbeing between carers and non-carers across different time points (from March 2020 to July/August 2021) during the COVID-19 pandemic using data from England. Results showed that informal carers experienced higher levels of depressive and anxiety symptoms than people without caring responsibilities across much of the pandemic. The relationship between being a carer and poorer mental health (particularly for depressive symptoms) was strongest during first and second lockdown and when all restrictions eased in summer 2021. Results were consistent when the sample was restricted to those who reported their mental health being adversely affected by the pandemic. Such differences were independent of socio-demographic backgrounds and personal characteristics, long-term health conditions, and stress about the virus or virus diagnosis. There was no evidence that carers differed from non-carers in loneliness and life satisfaction. However, we found that carers experienced a greater sense of their lives being worthwhile at the beginning of the first lockdown in England, but no difference was found at later time points when the lockdown measures were eased or when new restrictions were introduced. Among people with informal caring responsibilities, those who worked for 3 or more hours a day experienced greater anxiety symptoms when the first lockdown began to ease in comparison to carers who worked for less hours. No difference was found for other outcomes.

Our findings that carers had generally higher levels of depressive and anxiety symptoms during the COVID-19 pandemic are consistent with existing literature before the pandemic highlighting the mental health burden of informal caring^5,7^ and with qualitative and small-scale cross-sectional studies during the COVID-19 pandemic.^12–14,23^ The negative effect of caregiving can be explained by the chronic stress model. Care provision creates physical and psychological strain over extended periods of time, which is accompanied by high levels of unpredictability and uncontrollability, frequently requires high levels of vigilance, and creates secondary stress due to competing demands in other roles.^5^ Chronic stress can lead to psychosocial distress and worsening mental health. The negative experiences associated with caregiving were likely to intensify during the COVID-19 pandemic as a result of cuts to formal care, reduced paid working hours, reduced informal support from other relatives or friends, restricted access to healthcare services, and fear of virus infection.^12,14,15,24^ These experiences could be further exacerbated as the intensity for informal caring increased, as demonstrated in our sensitivity analysis that carers who worked for longer hours were more likely to feel anxious.

In addition to this, our study goes beyond previous finding by showing that the differences in depressive and anxiety symptoms between carers versus non-carers were fairly stable across the different stages and intensities of the lockdown restrictions. Levels of depressive symptoms continued to be higher among carers even when all COVID-19 related restrictions were lifted. There are a number of potential explanations for this. First, it could be explained by a feeling of exhaustion from the ongoing responsibilities lasting over a year. It is also possible that informal carers may have had greater concerns about the relaxation of restrictions, perhaps due to concerns about them or the person they cared for being more at risk again of coming into contact with the virus. It is further possible that with the relaxations, any additional support carers were receiving from friends or other relatives may have decreased as people had more opportunities to engage in usual leisure pursuits. While these results are not especially surprising,^39,40^ they are still of particular concern in the context of the pandemic as they suggest that, unlike for the general public,^41^ carers’ poorer mental health may be less likely to improve even when the lockdown measures were relaxed. Many vulnerable individuals have been more reliant than ever before on their informal carers. So if poor mental health leads to carer burnout, either affecting care during the pandemic or the willingness and capacity to provide care in the aftermath of the pandemic, this could have substantial implications for those individuals but also for the wider health and social care sector, leaving more work to be carried out by formal carers. In light of this, it is critical that informal carers are provided with adequate targeted mental health support.

Our results also provide some greater nuance in our understanding of specific aspects of carer mental health. First, it is notable that anxiety symptoms were only slightly (and not significantly) higher among carers than non-carers during first lockdown. This resonates with research showing a general increase in anxiety among the population as a whole when the pandemic first started, which may have led to a diminishing of the usually reported difference in anxiety among carers versus non-carers.^42^ At the same time, our study has shown that carers may also have experienced a greater sense of life being worthwhile compared to non-carers in the early part of the pandemic. This is in line with previous studies that show the positive experience of caregiving, such as gratification, companionship, meaning, sense of purpose, personal growth, and so forth.^9–11^ Our findings on worthwhileness provide empirical support for the view that both negative and positive experiences may emerge as independent dimensions as a result of caregiving.^43^ However, it is important to note that the difference in worthwhileness between carers and non-carers was only significant at the beginning of the lockdown. A potential explanation is that as the difficult situation unfolded, the initial greater sense of being worthwhile and appreciation by those they were caring for and others within communities may have been gradually eroded by the stresses of providing that care but also by the decreasing social recognition of the role carers were playing during the COVID-19 pandemic. Similar patterns have been noted for formal carers, who experienced greater societal appreciation in the early part of the pandemic (including with the national ‘clap for carers’) but who simultaneously reported decreasing appreciation from the government as the pandemic continued contributing to poorer morale.^44^ Furthermore, it is notable that carers still had higher depressive symptoms at the start of the pandemic, suggesting that this period still took a psychological toll.

It is also notable that we found no evidence that carers differ from non-carers in loneliness and life satisfaction, which seems to contradict to previous studies that show the correlation between being an informal carer and higher levels of loneliness and lower levels of life satisfaction (although results on life satisfaction are less conclusive as it varies across the types of care, the health conditions of the care recipients, the length of care, etc.).^15,43,45,46^ Previous studies have suggested that the reasons for these higher levels of loneliness and lower levels of wellbeing are that care provision is a time and energy consuming task that can restrict carers’ personal and social life. Indeed, a report from Carers UK showed that nearly half of the carers reported not having time to spend on social activities and difficulties being able to leave the house.^46^ However, such feelings may have changed in the context of the COVID-19 pandemic, although reports suggest loneliness and social isolation remained a challenge for many carers.^15^ Due to the lockdown and social distancing measures, face-to-face social activities were greatly restricted for the whole population. As a consequence, caring responsibilities may have reduced feelings of isolation among carers as others experienced some of the same social restrictions that they faced before the pandemic, and carers may have felt less of a sense of missing out. As carers had some exemptions from the ‘stay at home’ orders to visit the people they cared for, they might also have been able to maintain companionship during these difficult times. This is supported by a report showing that two in five young carers and one in five young adult carers built a stronger relationship with the person they were caring for during the pandemic^22^ and nearly three in five carers reported being able to keep in touch with family and friends despite the lockdown measures.^15^ It is also possible that the gap in the levels of loneliness and life satisfaction between carers and non-carers was reduced as a study has shown that mental health has worsened for the general population in the UK.^19^

This study had several limitations. First of all, the UCL COVID-19 Social Study did not use a random sample, therefore our sample is not representative of the population. However, the study does have a large sample size with wide heterogeneity, including good stratification across all major socio-demographic groups, and analyses were weighted based on population estimates of core demographics, with the weighted data showing good alignment with national population statistics and another large-scale nationally representative social survey.^47^ But we cannot rule out the possibility that the study inadvertently attracted individuals experiencing more extreme psychological experiences, with subsequent weighting for demographic factors failing to fully compensate for these differences. Moreover, like many other longitudinal studies, attrition remains an issue in our study and hence there was heterogeneity in our samples across the six time points. Second, the UCL COVID-19 Social Study did not collect any information before the pandemic. Therefore, we were not able to compare the average treatment effect of being a carer before and during the pandemic. Further work is needed to understand if the pandemic has heightened the mental health risk for carers compared with usual times. Third, this study treated carer status as a binary variable, without further exploring the intensity of caregiving (although a sensitivity analysis was run for the first few months of the pandemic), which has important implications for carers’ mental health and wellbeing. It is unknown whether individuals took on new informal caring responsibilities during the pandemic or withdrew from usual informal caring roles. Therefore, future work is needed to examine the role of care intensity and how fluctuating patterns of care affected mental health.^14^ Relatedly, while PSM can effectively control for observed confounding factors and can stimulate an experimental study on an observational dataset where an experimental setting is not feasible, it is unable to capture unobserved confounding factors. Therefore, future studies are needed to ascertain how experiences of carers versus non-carers varied depending on the type of care provided, the quality of the relationship between carers and the care recipients, and the health conditions of the care recipients. Finally, our analysis focused on comparisons between carers and non-carers at different time points in the pandemic, using PSM to control for confounding variables. However, this analysis did not show how the trajectories of mental health and wellbeing changed for carers versus non-carers, and this topic could be the focus of future research.

## Conclusion

The severe lockdown and social distancing measures implemented to control the spread of Covid-19 led to increasing burden for informal carers. The results of this study support some previous literature suggesting that carers were more likely to experience higher levels of depressive and anxiety symptoms during the pandemic, as in non-pandemic circumstances. But they build on these findings by quantifying this difference and showing how the mental health experiences changed in line with changing social restrictions during COVID-19. Carers were also more likely to feel a higher sense of life being worthwhile compared to non-carers, but this effect was attenuated after the first lockdown. In contrast to the existing studies, we found no differences in loneliness and life satisfaction between carers and non-carers, suggesting either that the companionship provided through caring during lockdown and social solidarity in experiencing social restrictions may have offered some emotional benefits to carers, or that worsening levels of personal and social wellbeing among non-carers (as documented in previous studies) closed the gap between the experiences of carers and non-carers. As carers are an important support to the national healthcare support, it is therefore crucial to integrate their needs into healthcare planning and delivery, especially when the health service is stretched as during this pandemic. While there is some existing support available to carers, the results presented here highlight the importance of ensuring adequate and targeted mental health provision to support carers during and following this pandemic so that they are able to continue their vital work.

## Supplemental Material

sj-docx-1-rsh-10.1177_17579139221104973 – Supplemental material for Mental health and wellbeing among people with informal caring responsibilities across different time points during the COVID-19 pandemic: a population-based propensity score matching analysis Supplemental material, sj-docx-1-rsh-10.1177_17579139221104973 for Mental health and wellbeing among people with informal caring responsibilities across different time points during the COVID-19 pandemic: a population-based propensity score matching analysis by Hei Wan Mak, Feifei Bu and Daisy Fancourt in Perspectives in Public Health
