# Supplementary material for: Mental health and wellbeing among people with informal caring responsibilities across different time points during the COVID-19 pandemic: a population-based propensity score matching analysis
Source: Perspect Public Health. 2022 Jul 5;143(5):275–84. doi: 10.1177/17579139221104973 (PMC10576404; doi:10.1177/17579139221104973)
Supplement: sj-docx-1-rsh-10.1177_17579139221104973 – Supplemental material for Mental health and wellbeing among people with informal caring responsibilities across different time points during the COVID-19 pandemic: a population-based propensity score matching analysis [file sj-docx-1-rsh-10.1177_17579139221104973.docx]

# Supplementary

| STable 1 Comparison of items in the original and revised Perceived Social Support Questionnaire (F-SozU K-6) | |
| --- | --- |
| Original | **Adapted for COVID-19**  **In the past week, I feel…** |
| I experience a lot of understanding and security from others | I have experienced a lot of understanding and support from others |
| I know a very close person whose help I can always count on | I have a very close person whose help I can always count on |
| If necessary, I can easily borrow something I might need from neighbours or friends | If necessary, I can easily borrow something I need from neighbours or friends |
| I know several people with whom I like to do things | I have people with whom I can spend time and do things together |
| When I am sick, I can without hesitation ask friends and family to take care of  important matters for me | If I get sick, I have friends and family who will take care of me |
| If I am down, I know to whom I can go without hesitation | If I am feeling down, I have people I can talk to without hesitation |


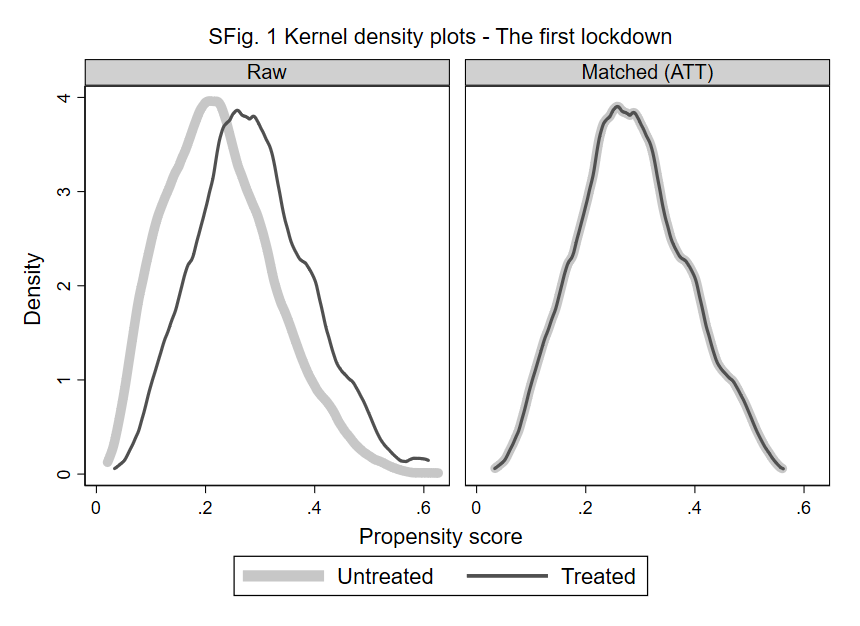

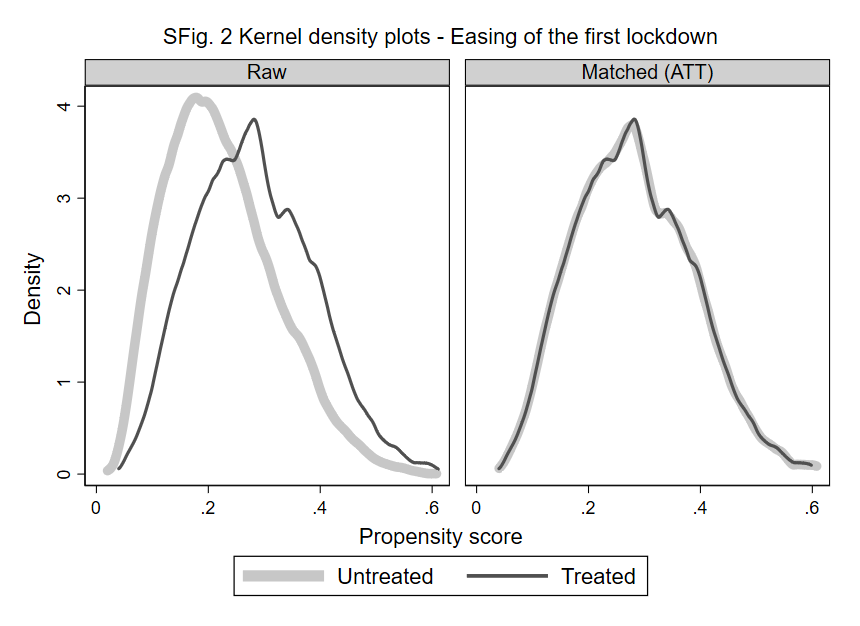

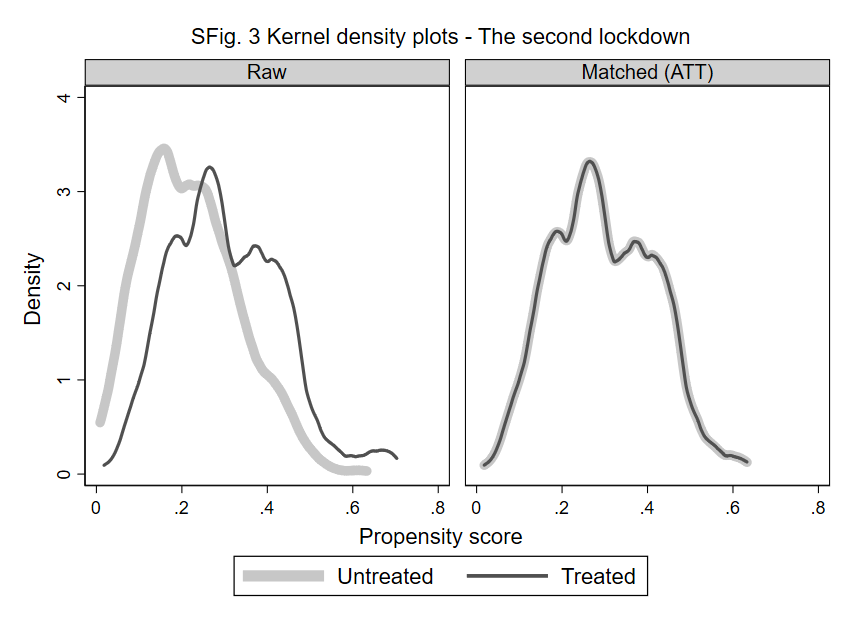

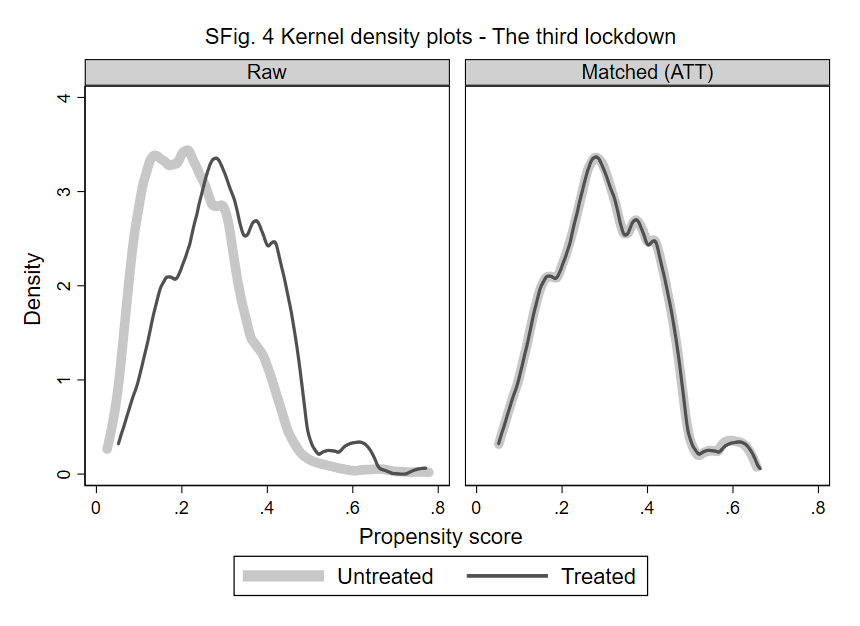

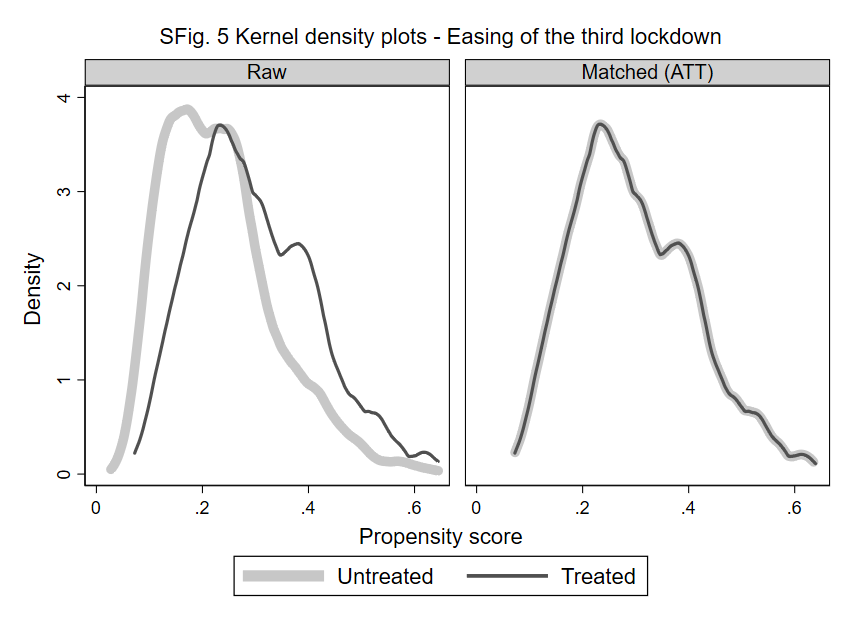

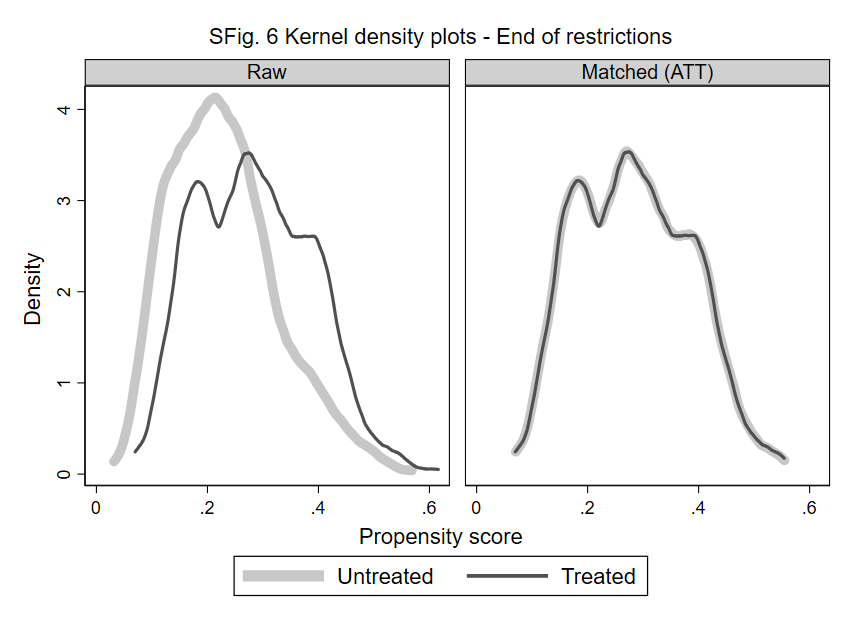


| **STable 2 Descriptive statistics of the sample from weighted data** | | | | | |  | |  | |  |  | |
| --- | --- | --- | --- | --- | --- | --- | --- | --- | --- | --- | --- | --- |
|  | | | First lockdown (28 March - 3 April 2020) | Easing of the first lockdown (16-22 May 2020) | | Second lockdown (14-20 November 2020) | | Third lockdown (16-22 January 2021) | | Easing of the third lockdown (20-26 March 2021) | End of restrictions (31 July – 6 August 2021) | |
|  | | | %/Mean(SE) | %/Mean(SE) | | %/Mean(SE) | | %/Mean(SE) | | %/Mean(SE) | %/Mean(SE) | |
| Carer | | | 24.2% | 23.7% | | 24.2% | | 24.7% | | 24.9% | 23.0% | |
| Non-carer | | | 75.8% | 76.3% | | 75.8% | | 75.3% | | 75.1% | 77.1% | |
| Age 18-29 | | | 8.15% | 7.95% | | 5.16% | | 5.52% | | 6.14% | 4.51% | |
| Age 30-59 | | | 45.0% | 45.1% | | 43.4% | | 41.7% | | 42.3% | 41.5% | |
| Age 60+ | | | 46.9% | 46.9% | | 51.5% | | 52.8% | | 51.5% | 54.0% | |
| Female | | | 50.0% | 50.6% | | 51.5% | | 49.5% | | 51.1% | 52.2% | |
| Male | | | 50.0% | 49.4% | | 48.5% | | 50.5% | | 48.9% | 47.8% | |
| White | | | 91.9% | 92.4% | | 93.6% | | 94.5% | | 92.4% | 94.2% | |
| Ethnic minority | | | 8.08% | 7.63% | | 6.39% | | 5.51% | | 7.57% | 5.78% | |
| Living alone | | | 22.3% | 22.6% | | 24.1% | | 24.4% | | 24.1% | 25.6% | |
| Not living along & not living with children | | | 60.0% | 59.0% | | 60.3% | | 59.7% | | 59.4% | 60.4% | |
| Not living along & living with children | | | 17.8% | 18.4% | | 15.6% | | 16.0% | | 16.5% | 14.0% | |
| Married or in a relationship | | | 68.6% | 68.0% | | 68.2% | | 66.9% | | 67.6% | 66.7% | |
| Not married or not in a relationship | | | 31.4% | 32.1% | | 31.8% | | 33.1% | | 32.4% | 33.3% | |
| Degree or above | | | 37.7% | 37.0% | | 37.4% | | 35.6% | | 36.5% | 36.2% | |
| Without a degree | | | 62.3% | 63.0% | | 62.6% | | 64.4% | | 63.5% | 63.8% | |
| Employed | | | 52.6% | 52.7% | | 50.3% | | 48.3% | | 47.6% | 48.1% | |
| Not employed | | | 47.5% | 47.4% | | 49.7% | | 51.7% | | 52.4% | 51.9% | |
| Household income <£30,000 | | | 48.3% | 48.1% | | 48.3% | | 50.7% | | 49.9% | 50.0% | |
| Household income ≥£30,000 | | | 51.8% | 51.9% | | 51.8% | | 49.3% | | 50.1% | 50.0% | |
| Keyworker | | | 18.5% | 18.9% | | 18.1% | | 16.4% | | 16.9% | 16.8% | |
| Non-keyworker | | | 81.5% | 81.1% | | 81.9% | | 83.6% | | 83.1% | 83.2% | |
| Living in a city/town | | | 78.0% | 77.9% | | 77.5% | | 77.9% | | 77.4% | 76.0% | |
| Living in a remote area (e.g. village/hamlet/isolated dwelling) | | | 22.0% | 22.1% | | 22.5% | | 22.1% | | 22.6% | 24.1% | |
| Long-term mental/physical health diagnosis | | | 52.4% | 53.1% | | 53.3% | | 53.4% | | 54.0% | 52.7% | |
| No long-term mental/physical health diagnosis | | | 47.7% | 46.9% | | 46.7% | | 46.6% | | 46.0% | 47.3% | |
| Having minor/major stress about COVID-19 | | | 55.9% | 43.3% | | 43.2% | | 46.8% | | 31.8% | 30.1% | |
| Not having minor/major stress about COVID-19 | | | 44.1% | 56.7% | | 56.8% | | 53.2% | | 68.2% | 69.9% | |
| Confirmed/suspected COVID-19 diagnosis | | | 8.08% | 11.2% | | 4.35% | | 4.12% | | 2.50% | 2.96% | |
| Not confirmed/suspected COVID-19 diagnosis | | | 91.9% | 88.8% | | 95.7% | | 95.9% | | 97.5% | 97.0% | |
| Perceived social support, ranging from 6-30 | | | 22.2 (0.09) | 21.9 (0.08) | | 22.6 (0.18) | | 22.3 (0.19) | | 22.7 (0.18) | 23.0 (0.20) | |
| Empathy – perspective-taking, ranging from 1-5 | | | 3.22 (0.01) | 3.22 (0.01) | | 3.21 (0.01) | | 3.21 (0.01) | | 3.23 (0.01) | 3.21 (0.01) | |
| Empathy – empathetic concern, ranging from 1-5 | | | 3.10 (0.01) | 3.11 (0.00) | | 3.11 (0.01) | | 3.10 (0.01) | | 3.11 (0.01) | 3.11 (0.01) | |
| Depressive symptoms, ranging from 0-27 | | | 5.51 (0.08) | 5.48 (0.07) | | 4.77 (0.14) | | 5.14 (0.18) | | 5.06 (0.13) | 4.19 (0.14) | |
| Anxiety symptoms, ranging from 0-21 | | | 4.47 (0.07) | 3.86 (0.06) | | 3.40 (0.11) | | 3.73 (0.15) | | 3.67 (0.12) | 3.12 (0.13) | |
| Loneliness, ranging from 3-9 | | | 4.66 (0.03) | 4.78 (0.02) | | 4.72 (0.05) | | 4.78 (0.05) | | 4.72 (0.05) | 4.49 (0.05) | |
| Life satisfaction, ranging from 0-10 | | | 6.25 (0.04) | 6.21 (0.03) | | 6.49 (0.06) | | 6.26 (0.07) | | 6.50 (0.06) | 7.16 (0.06) | |
| Sense of being worthwhile, ranging from 0-10 | | | 5.94 (0.03) | 6.06 (0.03) | | 6.18 (0.06) | | 5.91 (0.07) | | 6.38 (0.06) | 7.10 (0.06) | |
| **Observations** | | | **10,414** | **19,259** | | **3,712** | | **3,408** | | **4,068** | **3,128** | |
| **STable 3: Propensity score matching estimating the association between caring responsibilities and mental health/wellbeing across 6 different time-points during the COVID-19 pandemic** | | | | | | | | | | | |  |
|  | **Depressive symptoms** | **Anxiety symptoms** | | | **Loneliness** | | **Life satisfaction** | | **Sense of being worthwhile** | | |  |
| **First lockdown (28/03/20-03/04/20)** | | | | | | | | | | | |  |
| ATT (95%CI) | 0.45 (0.12, 0.78) ** | 0.27 (-0.03, 0.57) | | | -0.05 (-0.14, 0.05) | | -0.04 (-0.19, 0.11) | | 0.29 (0.14, 0.44)*** | | |  |
| Control group | 7,771 | | | | | | | | | | |  |
| Treatment group | 2,535 | | | | | | | | | | |  |
| Total N | 10,306 | | | | | | | | | | |  |
| **Easing of the first lockdown (16/05/20-22/05/20)** | | | | | | | | | | | |  |
| ATT (95%CI) | 0.55 (0.27, 0.84) *** | 0.42 (0.17, 0.67)** | | | 0.02 (-0.06, 0.10) | | -0.04 (-0.15, 0.07) | | 0.11 (-0.01, 0.23) | | |  |
| Control group | 14,633 | | | | | | | | | | |  |
| Treatment group | 4,612 | | | | | | | | | | |  |
| Total N | 19,245 | | | | | | | | | | |  |
| **Second lockdown (14/11/20-20/11/20)** | | | | | | | | | | | |  |
| ATT (95%CI) | 0.78 (0.18, 1.38)* | 0.84 (0.33, 1.35)** | | | 0.08 (-0.10, 0.26) | | -0.26 (-0.54, 0.02) | | -0.15 (-0.44, 0.13) | | |  |
| Control group | 2,729 | | | | | | | | | | |  |
| Treatment group | 927 | | | | | | | | | | |  |
| Total N | 3,656 | | | | | | | | | | |  |
| **Third lockdown (16/01/21-22/01/21)** | | | | | | | | | | | |  |
| ATT (95%CI) | 0.70 (-0.06, 1.46) | 0.77 (0.05, 1.49)* | | | 0.06 (-0.14, 0.26) | | 0.09 (-0.18, 0.36) | | 0.22 (-0.14, 0.59) | | |  |
| Control group | 2,557 | | | | | | | | | | |  |
| Treatment group | 825 | | | | | | | | | | |  |
| Total N | 3,382 | | | | | | | | | | |  |
| **Easing of the third lockdown (20/03/21-26/03/21)** | | | | | | | | | | | |  |
| ATT (95%CI) | -0.09 (-0.76, 0.58) | 0.10 (-0.50, 0.70) | | | -0.06 (-0.24, 0.12) | | 0.13 (-0.09, 0.35) | | 0.16 (-0.08, 0.40) | | |  |
| Control group | 3,012 | | | | | | | | | | |  |
| Treatment group | 1,017 | | | | | | | | | | |  |
| Total N | 4,029 | | | | | | | | | | |  |
| **End of restrictions (31/07/21-06/08/21)** | | | | | | | | | | | |  |
| ATT (95%CI) | 1.01 (0.44, 1.59)** | 0.62 (0.06, 1.17)* | | | 0.07 (-0.10, 0.25) | | -0.15 (-0.40, 0.09) | | -0.03 (-0.29, 0.23) | | |  |
| Control group | 2,374 | | | | | | | | | | |  |
| Treatment group | 734 | | | | | | | | | | |  |
| Total N | 3,108 | | | | | | | | | | |  |

| **STable 4 Propensity score matching: amongst respondents who reported mental health had got worse during the first lockdown in April/May vs pre-pandemic** | | | | | |
| --- | --- | --- | --- | --- | --- |
|  | **Depressive symptoms** | **Anxiety symptoms** | **Loneliness** | **Life satisfaction** | **Sense of being worthwhile** |
| **First lockdown (28/03/20-03/04/20)** | | | | | |
| ATT (95%CI) | 0.70 (-0.08, 1.47) | 0.50 (-0.17, 1.16) | -0.04 (-0.25, 0.18) | -0.08 (-0.40, 0.24) | 0.17 (-0.17, 0.51) |
| Control group | 2,153 | | | | |
| Treatment group | 735 | | | | |
| Total N | 2,888 | | | | |
| **Easing of the first lockdown (16/05/20-22/05/20)** | | | | | |
| ATT (95%CI) | 0.58 (-0.10, 1.26) | 0.58 (-0.05, 1.21) | -0.02 (-0.21, 0.17) | 0.04 (-0.18, 0.25) | 0.19 (-0.04, 0.43) |
| Control group | 4,196 | | | | |
| Treatment group | 1,402 | | | | |
| Total N | 5,598 | | | | |
| **Second lockdown (14/11/20-20/11/20)** | | | | | |
| ATT (95%CI) | 0.43 (-1.01, 1.87) | 0.74 (-0.49, 1.96) | 0.11 (-0.24, 0.46) | -0.06 (-0.55, 0.44) | -0.02 (-0.54, 0.50) |
| Control group | 814 | | | | |
| Treatment group | 274 | | | | |
| Total N | 1,088 | | | | |
| **Third lockdown (16/01/21-22/01/21)** | | | | | |
| ATT (95%CI) | 0.58 (-0.91, 2.07) | 1.19 (-0.35, 2.74) | -0.30 (-0.71, 0.12) | 0.26 (-0.28, 0.81) | 0.55 (-0.35, 1.46) |
| Control group | 679 | | | | |
| Treatment group | 263 | | | | |
| Total N | 942 | | | | |
| **Easing of the third lockdown (20/03/21-26/03/21)** | | | | | |
| ATT (95%CI) | -0.20 (-1.58, 1.17) | -0.01 (-1.33, 1.31) | -0.23 (-0.60, 0.14) | 0.45 (-0.06, 0.95) | 0.33 (-0.18, 0.84) |
| Control group | 858 | | | | |
| Treatment group | 292 | | | | |
| Total N | 1,150 | | | | |
| **End of restrictions (31/07/21-06/08/21)** | | | | | |
| ATT (95%CI) | 1.93 (0.51, 3.35)** | 1.05 (-0.43, 2.52) | 0.08 (-0.27, 0.43) | -0.39 (-0.93, 0.16) | -0.34 (-0.98, 0.29) |
| Control group | 636 | | | | |
| Treatment group | 214 | | | | |
| Total N | 850 | | | | |

| **STable 5a Propensity score matching comparing caring intensity amongst informal carers: cared for 3 or more hours a day vs less than 3 hours** | | | | | |
| --- | --- | --- | --- | --- | --- |
|  | **Depressive symptoms** | **Anxiety symptoms** | **Loneliness** | **Life satisfaction** | **Sense of being worthwhile** |
| **First lockdown (28/03/20-03/04/20)** | | | | | |
| ATT (95%CI) | 0.09 (-0.92, 1.10) | 0.24 (-0.61, 1.09) | -0.06 (-0.32, 0.20) | -0.12 (-0.50, 0.27) | 0.09 (-0.29, 0.48) |
| Control group | 1,060 | | | | |
| Treatment group | 557 | | | | |
| Total N | 1,617 | | | | |
| **Easing of the first lockdown (16/05/20-22/05/20)** | | | | | |
| ATT (95%CI) | 0.37 (-0.38, 1.12) | 0.69 (0.10, 1.28)* | 0.10 (-0.10, 0.31) | -0.07 (-0.33, 0.19) | 0.17 (-0.09, 0.44) |
| Control group | 1,610 | | | | |
| Treatment group | 1,008 | | | | |
| Total N | 2,618 | | | | |

| **STable 5b Propensity score matching comparing caring intensity amongst informal carers: cared for 6 or more hours a day vs less than 6 hours** | | | | | |
| --- | --- | --- | --- | --- | --- |
|  | **Depressive symptoms** | **Anxiety symptoms** | **Loneliness** | **Life satisfaction** | **Sense of being worthwhile** |
| **First lockdown (28/03/20-03/04/20)** | | | | | |
| ATT (95%CI) | 0.54 (-0.37, 1.44) | 0.71 (-0.07, 1.49) | -0.00 (-0.25, 0.24) | 0.29 (-0.10, 0.68) | -0.06 (-0.41, 0.29) |
| Control group | 1,295 | | | | |
| Treatment group | 417 | | | | |
| Total N | 1,712 | | | | |
| **Easing of the first lockdown (16/05/20-22/05/20)** | | | | | |
| ATT (95%CI) | 0.44 (-0.36, 1.23) | 0.72 (0.05, 1.38)* | 0.18 (-0.04, 0.41) | 0.01 (-0.30, 0.32) | -0.20 (-0.49, 0.08) |
| Control group | 1,922 | | | | |
| Treatment group | 700 | | | | |
| Total N | 2,622 | | | | |
